# Postmenopausal bleeding associated with semaglutide: a diagnostic challenge

**DOI:** 10.1210/jcemcr/luag261

**Published:** 2026-09-16

**Authors:** Sai Prasad, Vidhi Parmar, Anukriti Sharma, Navjot Kaur, Darpan Kothia, Deep Bavaria

**Affiliations:** Department of Internal Medicine, S. Nijalingappa Medical College, Navanagar, Bagalkote, Karnataka 587103, India; Department of Endocrinology, Mayo Clinic, Rochester, MN 55905, USA; Department of Internal Medicine, Jaipur National University Institute of Medical Sciences and Research Centre, Jaipur, Rajasthan 302017, India; Department of Internal Medicine, Government Medical College Patiala, Patiala, Punjab 147001, India; Department of Internal Medicine, Ascension Sacred Heart Pensacola, Florida State University, Pensacola, FL 32504, USA; Department of Internal Medicine, MP Shah Government Medical College, Jamnagar, Gujarat 361006, India

**Keywords:** semaglutide, GLP-1 receptor agonist, postmenopausal bleeding, adverse drug reaction, endometrial instability

## Abstract

Semaglutide, a glucagon-like peptide-1 (GLP-1) receptor agonist, is widely used for obesity and type 2 diabetes management, but its gynecological adverse effects have not been systematically described. We report a 58-year-old postmenopausal woman with new-onset vaginal bleeding six weeks after starting semaglutide for weight reduction, with onset following dose escalation to 0.5 mg weekly. Hemoglobin fell from 12.9 g/dL (Système International [SI]: 129 g/L) to 10.8 g/dL (SI: 108 g/L) (reference range, 12.0-16.0 g/dL [SI: 120-160 g/L]). The serum follicle-stimulating hormone (FSH) concentration was elevated at 68.4 mIU/mL (SI: 68.4 IU/L; reference, >25 IU/L postmenopausal), and estradiol was suppressed at 3.5 pg/mL (SI: 13 pmol/L; reference, <5.4 pg/mL [SI: <20 pmol/L]), consistent with stable postmenopausal status throughout, without hormonal reactivation (no return toward a premenopausal pattern). Endometrial malignancy, structural pathology, and coagulopathy were excluded. The Naranjo Adverse Drug Reaction Probability Scale score was 6 (“probable”). Bleeding resolved completely within five weeks of discontinuation. We propose that rapid semaglutide-induced adipose loss may transiently alter peripheral estrogen metabolism, destabilizing the endometrium despite unchanged hormone levels. This case highlights postmenopausal bleeding as a rare but clinically important adverse effect of GLP-1 receptor agonist therapy warranting malignancy-first evaluation.

## Introduction

Semaglutide is a long-acting glucagon-like peptide-1 (GLP-1) receptor agonist approved for the treatment of type 2 diabetes mellitus and, at higher doses, for chronic weight management [1]. Its mechanism of action includes augmentation of glucose-dependent insulin secretion, suppression of glucagon release, delayed gastric emptying, and reduction of appetite through central hypothalamic pathways [2]. Its expanding use has been accompanied by growing attention to its adverse effect profile, which includes well-documented gastrointestinal effects such as nausea, vomiting, diarrhea, and constipation [3].

Postmenopausal bleeding is defined as vaginal bleeding occurring 12 or more months after the final menstrual period and is a significant clinical concern given its association with endometrial malignancy in approximately 10% of cases [4]. The differential diagnosis is broad and includes endometrial atrophy, polyps, hyperplasia, carcinoma, cervical pathology, exogenous estrogen exposure, coagulopathy, medication-induced bleeding, and hormonal reactivation [5]. Hormonal reactivation refers to a return toward a premenopausal pattern of gonadotropin and estradiol secretion, suggestive of resumed ovarian follicular activity, a phenomenon rarely documented after true menopause [6]; in this patient it was excluded, together with the other etiologies above, on comprehensive evaluation.

Adipose tissue is an important extragonadal source of estrogen in postmenopausal women through aromatase-mediated conversion of androstenedione to estrone [7]. A rapid reduction in adipose mass, as frequently observed with GLP-1 receptor agonist therapy, could theoretically alter peripheral estrogen metabolism and transiently destabilize the endometrium. To our knowledge, this is among the earliest reported cases of new-onset postmenopausal bleeding temporally and causally linked to semaglutide in the absence of demonstrable hormonal reactivation or structural pathology.

## Case presentation

A 58-year-old woman presented with a six-week history of vaginal bleeding. She had reached natural menopause at age 50 and had been amenorrheic for eight years, with no prior postmenopausal bleeding. Her medical history included obesity and prediabetes. Semaglutide was started eight weeks before presentation at 0.25 mg subcutaneously weekly for weight reduction, escalated to 0.5 mg weekly after four weeks. Spotting began approximately ten days after dose escalation and progressed to moderate daily bleeding over the following two weeks.

On examination, her height was 162 cm and weight 84.6 kg (body mass index [BMI] 32.2 kg/m^2^), reflecting a 7.8-kg reduction (8.4% of total body weight) from her pretreatment baseline of 92.4 kg over eight weeks. Vital signs were stable. Speculum examination showed no cervical lesions, active discharge, or severe vaginal atrophy that could account for the bleeding. Bimanual examination revealed no adnexal tenderness, uterine enlargement, or pelvic mass.

The patient denied hormone replacement therapy, selective estrogen receptor modulators, anticoagulants, phytoestrogens, or herbal preparations. There was no personal or family history of gynecological malignancy, coagulation disorders, or autoimmune endocrine disease. She reported mild lower abdominal discomfort and fatigue but denied fever, galactorrhea, vasomotor symptoms, trauma, or recent intercourse.

## Diagnostic assessment

Aside from a hemoglobin obtained four months earlier, no pretreatment values were available, as the patient had no prior indication for gynecological or endocrine testing; Table 1 therefore reports values at presentation and follow-up, with the baseline hemoglobin given below.

**Table 1:** Summary of laboratory and diagnostic investigations

Investigation	At Presentation	At Follow-up	Reference Range	Interpretation
**Hematology**				
Hemoglobin*^a^*	10.8 g/dL (SI: 108 g/L)	11.9 g/dL (SI: 119 g/L), 6 wk post-discontinuation	12.0-16.0 g/dL (SI: 120-160 g/L)	Fall consistent with blood loss; improved after discontinuation
Platelet count	274 × 10^9^/L	Not repeated	150-400 × 10^9^/L	No thrombocytopenia
**Coagulation**				
Prothrombin time (PT)	12.4 s	Not repeated	11-14 s	No coagulopathy
Activated partial thromboplastin time (aPTT)	30.1 s	Not repeated	25-35 s	No coagulopathy
**Endocrine and Hormonal**				
Follicle-stimulating hormone (FSH)	68.4 mIU/mL (SI: 68.4 IU/L)	69.1 mIU/mL (SI: 69.1 IU/L)	>25 mIU/mL (SI: >25 IU/L) (postmenopause)	Persistently elevated; no reactivation
Luteinizing hormone (LH)	34.8 mIU/mL (SI: 34.8 IU/L)	35.2 mIU/mL (SI: 35.2 IU/L)	>15 mIU/mL (SI: >15 IU/L) (postmenopause)	Stable postmenopausal status
Estradiol (E2)	3.5 pg/mL (SI: 13 pmol/L)	3.3 pg/mL (SI: 12 pmol/L)	<5.4 pg/mL (SI: <20 pmol/L)	Suppressed; no reactivation
Thyroid-stimulating hormone (TSH)	2.03 mIU/L	Not repeated	0.4-4.0 mIU/L	No thyroid dysfunction
Prolactin	10.6 ng/mL (SI: 10.6 µg/L)	Not repeated	<25 ng/mL (SI: <25 µg/L)	No hyperprolactinemia
Serum beta-hCG	<2 mIU/mL (SI: <2 IU/L)	Not repeated	<5 mIU/mL (SI: <5 IU/L)	Pregnancy excluded
**Biochemistry**				
Liver function tests	Normal	Not repeated	Within laboratory reference	No hepatic dysfunction
**Microbiology**				
Vaginal swab microscopy and culture	No growth/no pathogens	Not repeated	Negative	Infectious etiology excluded
**Gynecological Investigations**				
Cervical cytology	Negative for intraepithelial lesion or malignancy (6 mo before presentation)	Not repeated	Normal (NILM)	Cervical pathology excluded
Transvaginal ultrasonography	Thin endometrium; no focal abnormality	Not repeated	Endometrial thickness <4 mm (postmenopause)	No structural pathology identified
Endometrial biopsy (histopathology)	Inactive, atrophic endometrium	Not repeated	No hyperplasia or malignancy	Endometrial malignancy and hyperplasia excluded
**Causality Assessment**				
Naranjo ADR Probability Scale	Score: 6	Not repeated	≥9 Definite; 5-8 Probable; 1-4 Possible; ≤0 Doubtful	Probable adverse drug reaction to semaglutide

^*a*^Baseline (pretreatment) hemoglobin, obtained 4 months before treatment initiation, was 12.9 g/dL (SI: 129 g/L). Pretreatment values for other analytes were not available, as the patient had no clinical indication for gynecological or endocrine testing prior to symptom onset.

Abbreviations: ADR, adverse drug reaction; NILM, negative for intraepithelial lesion or malignancy; SI, Système International.

Laboratory investigations, summarized in Table 1, showed a hemoglobin of 10.8 g/dL (Système International [SI] units: 108 g/L), reduced from a baseline of 12.9 g/dL (SI: 129 g/L) obtained four months earlier, consistent with normocytic anemia from ongoing blood loss. Coagulation studies were normal: prothrombin time (PT) was 12.4 seconds (reference range, 11-14 seconds) and activated partial thromboplastin time (aPTT) was 30.1 seconds (reference range, 25-35 seconds), as was the platelet count, 274 × 10^9^/L (reference range, 150-400 × 10^9^/L), excluding a primary bleeding diathesis. Thyroid-stimulating hormone (TSH) was 2.03 mIU/L (reference range, 0.4-4.0 mIU/L), prolactin was 10.6 ng/mL (SI: 10.6 µg/L; reference, <25 ng/mL), and liver function tests were unremarkable.

The serum follicle-stimulating hormone (FSH) concentration was 68.4 mIU/mL (SI: 68.4 IU/L; postmenopausal range, >25 IU/L), and the serum estradiol concentration was 3.5 pg/mL (SI: 13 pmol/L; postmenopausal range, <5.4 pg/mL [SI: <20 pmol/L]); findings consistent with stable postmenopausal status, without hormonal reactivation. Luteinizing hormone (LH) was likewise elevated at 34.8 mIU/mL (SI: 34.8 IU/L; postmenopausal range, >15 IU/L). Serum beta-human chorionic gonadotropin (beta-hCG) was negative at <2 mIU/mL (SI: <2 IU/L; reference, <5 mIU/mL). These findings argue against spontaneous ovarian reactivation and exclude exogenous estrogen exposure as a contributing mechanism.

Transvaginal ultrasonography showed a thin endometrium (<4 mm) without focal abnormality. Cervical cytology performed six months earlier was negative for intraepithelial lesion or malignancy. Vaginal swab microscopy and culture were negative for bacterial vaginosis, Trichomonas vaginalis, and other pathogens. Endometrial biopsy, performed to exclude malignancy and hyperplasia, showed an inactive, atrophic endometrium without hyperplasia or neoplasia.

Given the absence of structural, infectious, hormonal, or hematological explanations and the clear temporal association between semaglutide dose escalation and symptom onset, a probable adverse drug reaction (ADR) was suspected. The Naranjo ADR Probability Scale was applied and yielded a score of 6, within the “probable” category, reflecting a clear temporal relationship, resolution of bleeding following drug discontinuation (“dechallenge”), and the absence of alternative etiologies on comprehensive evaluation; points for a positive rechallenge could not be scored, as the patient declined re-exposure.

## Treatment

Following multidisciplinary discussion involving endocrinology and gynecology, semaglutide was discontinued given its probable causal association with bleeding. Given hemodynamic stability and an atrophic endometrium on biopsy, conservative management was considered appropriate. Hormone therapy was withheld: because FSH, LH, and estradiol were all appropriate for her menopausal status, there was no biochemical estrogen deficiency to correct, and her bleeding was attributed to a probable adverse effect of semaglutide, most plausibly a transient alteration in peripheral estrogen exposure as discussed below, rather than to estrogen-deficiency-related atrophic bleeding.

Oral iron was started for symptomatic anemia. She was counseled on warning symptoms warranting immediate review, including hemodynamically significant bleeding, dizziness, or syncope. Structured dietary modification and supervised physical activity were discussed and begun as alternative weight-management strategies. Re-exposure to semaglutide was deferred jointly with the patient: we reasoned that a further period of rapid fat loss on rechallenge, rather than her absolute weight, could reproduce a similar transient estrogen flux, and this reasoning was discussed as part of shared decision-making.

## Outcome and follow-up

Within two weeks of discontinuation, bleeding frequency and volume markedly decreased, with complete cessation by five weeks. Fatigue improved in parallel with hematologic recovery, and a repeat hemoglobin at six weeks was 11.9 g/dL (SI: 119 g/L). Repeat hormonal testing at follow-up showed FSH 69.1 mIU/mL (SI: 69.1 IU/L), LH 35.2 mIU/mL (SI: 35.2 IU/L), and estradiol 3.3 pg/mL (SI: 12 pmol/L), each essentially unchanged from presentation and consistent with stable postmenopausal status without hormonal reactivation. Over four months of follow-up, no further bleeding or new gynecological or endocrine abnormalities occurred. The patient declined rechallenge, citing concern about recurrence, and continued gradual weight reduction through non-pharmacological means without adverse sequelae.

## Discussion

This case documents a probable adverse drug reaction: new-onset postmenopausal bleeding in close temporal association with semaglutide dose escalation, resolving fully on withdrawal, without an identifiable structural, infectious, coagulative, or hormonal alternative explanation.

Adipose tissue is the predominant source of endogenous estrogen in postmenopausal women via aromatase-mediated conversion of adrenal androstenedione [7], and greater adiposity is associated with higher circulating estrogen and endometrial cancer risk [8]; conversely, intentional weight loss lowers circulating estrogen and androgen concentrations in postmenopausal women [9, 10]. We propose that a rapid, substantial reduction in adipose mass, conceptually analogous to progestogen-withdrawal bleeding, could produce a transient withdrawal effect on estrogen-primed endometrium; her 7.8-kg loss (8.4% of baseline weight) over eight weeks fits this compressed timeframe.

A stimulatory mechanism, whether from peripheral estrogen or a direct endometrial effect, would be expected to produce a proliferative rather than atrophic endometrium, an apparent inconsistency worth addressing directly. Her biopsy and repeat hormone measurements, however, were obtained several weeks after bleeding onset, once any transient rise in peripheral estrogen exposure had presumably receded, analogous to how endometrium sampled after progestogen withdrawal reverts to an atrophic pattern once bleeding has occurred. The histology therefore likely reflects a post-withdrawal state rather than a preceding proliferative phase, and does not exclude an earlier transient stimulatory event. We regard estrogen withdrawal as the most parsimonious explanation; a direct proliferative GLP-1 receptor-mediated effect on the endometrium fits the atrophic histology less well and is discussed below as a hypothesis for future study rather than a confirmed contributor here.

GLP-1 receptors are expressed in the ovary, uterus, and endometrium, and preclinical data suggest GLP-1 signaling may influence endometrial proliferation [11], although this remains unconfirmed in humans and sits less comfortably with our patient's atrophic histology. GLP-1 receptor agonists have also been reported to influence the hypothalamic-pituitary-gonadal axis experimentally [12], raising the possibility of peripheral estrogen effects without measurable gonadotropin change.

The Naranjo ADR Probability Scale is a structured instrument for estimating the likelihood that a clinical event represents a genuine adverse drug reaction [13]; it assigns weighted points across criteria including temporal relationship, dechallenge/rechallenge response, and alternative etiologies. Documenting the score and its constituent criteria, rather than the total alone, allows causality assessments to be communicated transparently.

This adverse effect has not been systematically described in pivotal semaglutide trials such as SUSTAIN and STEP [3, 14], likely reflecting under-representation of older postmenopausal women, misattribution of bleeding to other causes, and underreporting in spontaneous surveillance systems. Real-world pharmacovigilance data have begun to capture this signal: a 2026 analysis found gynecological hemorrhagic events, including postmenopausal hemorrhage, reported in a small proportion of women using semaglutide and tirzepatide at comparable rates [15], and a separate case report described severe perimenopausal bleeding associated with dulaglutide, another GLP-1 receptor agonist [16]. Together with our case, these reports suggest endometrial and vaginal bleeding may represent a class effect of GLP-1 receptor agonists rather than an idiosyncratic reaction to semaglutide alone.

Postmenopausal bleeding in any patient requires thorough evaluation to exclude malignancy before attribution to a drug effect. Here, endometrial biopsy, transvaginal ultrasonography, and cervical cytology were all benign, allowing a probable adverse drug reaction to be diagnosed with reasonable confidence; the temporal sequence, resolution with dechallenge, and Naranjo score of 6 together support causality. As GLP-1 receptor agonist prescribing expands among older women, clinicians should remain alert to this presentation and consider pharmacovigilance reporting.

## Learning points

- Postmenopausal bleeding may be a rare adverse effect of semaglutide, plausibly mediated by rapid adipose loss altering peripheral estrogen metabolism.
- All new-onset postmenopausal bleeding requires comprehensive evaluation to exclude malignancy, structural pathology, and coagulopathy before attribution to a drug.
- Standard immunoassays may lack sensitivity for small hormonal fluctuations in postmenopausal women; LC-MS/MS offers greater accuracy at low estrogen concentrations in ambiguous cases [17].
- The Naranjo ADR Probability Scale is a useful tool for formalizing causality assessment and should be documented with its constituent criteria.
- As GLP-1 receptor agonist use expands, clinicians should remain vigilant for gynecological adverse effects and report such cases to pharmacovigilance registries.

## Contributors

SP and VP were directly involved in the clinical care, evaluation, and management of the patient. SP identified the case and takes responsibility for the overall content as guarantor. VP, AS, NK, DK, and DB contributed to clinical data interpretation, critical review of diagnostic findings, and development of the clinical discussion. All authors reviewed and approved the final version of the manuscript and agree to be accountable for all aspects of the work.

## Data Availability

Original data generated and analyzed for this case report are included in this published article.
